# Idiopathic Primary Hypoparathyroidism Presenting as Focal Seizures in a Neonate: A Rare Occurrence

**DOI:** 10.7759/cureus.10348

**Published:** 2020-09-10

**Authors:** Talal Almas, Irfan Ullah, Mehwish Kaneez, Maryam Ehtesham, Shahzad Rauf

**Affiliations:** 1 Internal Medicine, Royal College of Surgeons in Ireland, Dublin, IRL; 2 Researcher, Undergraduate Research Organisation, Dhaka, BGD; 3 Internal Medicine, Kabir Medical College, Peshawar, PAK; 4 Internal Medicine, Naseer Teaching Hospital, Peshawar, PAK; 5 Internal Medicine, Rawalpindi Medical University, Rawalpindi, PAK; 6 Pediatrics, Khyber Teaching Hospital, Peshawar, PAK

**Keywords:** neonatal hypoparathyroidism, focal seizures, hypocalcemia

## Abstract

Focal seizures in neonates presenting to the emergency department can be potentially dangerous and life-threatening. Hypoparathyroidism is an exceedingly rare but treatable cause of focal neonatal seizures. Due to its weak association with neonatal seizures, hypoparathyroidism often remains undetected as a potential cause. We hereby elucidate a rare case of idiopathic hypoparathyroidism presenting as right-sided focal seizures in a 12-day-old female neonate with an uneventful birth history. Despite the administration of antibiotics and phenobarbitone, the seizures remained unabated. Laboratory workup revealed the diagnosis of late-onset hypocalcemia with severe hypoparathyroidism. The patient was subsequently treated with calcium supplements and alfacalcidol. Thereafter, the frequency of seizures gradually decreased and the same treatment regimen was continued until the patient was seizure-free. The patient continues to do well to date with complete remission of his clinical symptoms.

## Introduction

Neonatal seizures, which are a frequently encountered medical emergency, can arise due to a multitude of clinical conditions [1]. Timely assessment and treatment of the etiology underlying neonatal seizures are pivotal [2]. Failure to detect the underlying cause of neonatal seizures can lead to adverse outcomes, including cognitive impairment, potentially culminating in death [1-2]. The presence of focal seizures in a neonate can be a sign of worsening meningitis, sepsis, cerebral ischemia, inherent metabolic errors, or electrolyte imbalance. Nevertheless, the presentation of endocrinological disorders in neonates as focal seizures is exceedingly rare [3-4]. We hereby delineate an interesting case of a 12-day-old female neonate who presented with right-sided focal fits. Initial laboratory workup revealed hypocalcemia, with all other electrolytes in their normal ranges. Further diagnostic workup revealed low levels of parathyroid hormone, thereby unmasking a diagnosis of idiopathic primary hypoparathyroidism presenting as late-onset hypocalcemic fits.

## Case presentation

A 12-day-old, full-term female baby, previously normal, was brought to the emergency department with right-sided focal fits. The baby was the product of a consanguineous marriage. However, consanguineous marriage might not be a cause of the patient's symptoms. The antenatal and birth histories of the baby were uneventful. Pertinently, the mother had no prior history of diabetes, hyperparathyroidism, hyperlipidemia, epilepsy, or any other endocrinological or metabolic disturbances during pregnancy that might be a cause of the baby's focal seizures. On arrival, the patient was vitally stable, active, and demonstrated no signs of dysmorphism. The occipitofrontal circumference (OFC) was 36 cm, the anterior fontanelle was normal, and the suckling reflex was intact. Physical examination revealed unremarkable hips, back, and genitalia. The lungs were clear on auscultation, with no audible murmur and no visceromegaly on abdominal examination. Thereafter, the patient was admitted to the special neonatal unit. Despite the administration of empirical intravenous antibiotics (cefotaxime and gentamicin), the patient continued to have right-sided focal fits for which she was given phenobarbitone. There was mild clinical improvement after phenobarbitone administration but the seizures remained unabated.

The laboratory investigations revealed hypocalcemia with hypoparathyroidism. Since low levels of parathyroid hormone are intricately linked with an increased incidence of hypocalcemia and increased seizure frequency, it was established as a cause of the patient's focal fits. Table 1 elucidates the patient's laboratory findings with their normal ranges.

**Table 1:** The patient’s laboratory workup results

Investigations	Results	Normal values
Hemoglobin	15 g/dL	13.4-19.9 g/dL
Total lymphocyte count	10,000/microlitre	4000-11,000/microliter.
Platelet count	368,000/microlitre	150,000-450,000/microliter
C-reactive protein	Normal	<7 mg/L
Total serum calcium	5.5 mg/dL	8.4-10.6 mg/dL
Parathyroid hormone	<1.2 pg/mL	14-67 pg/ml
Serum phosphorus	8.2 mg/dL	4.2-9.0 mg/dl
Serum magnesium	2.5 mg/dL	1.7 to 2.4 mg/dL
Alkaline phosphatase	429 U/L	48-406 U/L

To further evaluate the cause of the patient's hypoparathyroidism, a contrast-enhanced computed tomography (CT) scan of the head, neck, and chest was performed that revealed no abnormalities, with no evidence of Di-George syndrome. The laboratory workup alluded to the patient's underlying hypoparathyroidism as a potential cause of the patient’s hypocalcemic fits. Notably, most cases of hypoparathyroidism in neonates are due to maternal hyperparathyroidism, which suppresses the fetal parathyroid hormone levels and thus causes seizures [3-4]. Interestingly, the gestational maternal parathyroid hormone levels were in normal ranges (31-37 pg/ml during the pregnancy), yielding the diagnosis of idiopathic primary neonatal hypoparathyroidism. Therefore, all other causes of hypoparathyroidism such as Di-George syndrome, maternal hyperparathyroidism, and hypomagnesemia, were ruled out in our patient. Alfacalcidol, calcium carbonate, and calcium gluconate were thus added to the treatment regimen in order to correct the hypocalcemia and decrease the frequency of seizures in the patient. Thereafter, the frequency of the fits gradually decreased and the patient’s clinical symptoms abated.

## Discussion

Hypocalcemia consequent to low levels of circulating parathyroid hormone is the hallmark of hypoparathyroidism [5]. Hypoparathyroidism due to non-surgical causes, such as Di-George syndrome, idiopathic, or autoimmune destruction of parathyroid glands, is an exceedingly rare entity with a prevalence hovering around two to three per 100,000 adults; however, its prevalence in neonates is even lower, with only scarce data available [6]. The previously published literature espouses the notion that neonates with idiopathic primary hypoparathyroidism present with low levels of circulating calcium that might, in turn, lead to the development of seizures [3-4]. In our case, the etiology was suspected to be of an infectious nature on presentation, but the laboratory findings suggested otherwise. Intravenous infusion of cefotaxime and gentamicin was thus commenced; however, the frequency of the seizures remained unaltered. As a result, phenobarbitone was administered, owing to its anti-seizure activity, which subsequently resulted in clinical improvement.

Hypocalcemia in neonates is described as total serum calcium levels of less than 7 mg/dL [7]. Our patient presented to us with late-onset hypocalcemia, which is defined as hypocalcemia occurring after the first week of life. The causes of late-onset neonatal hypocalcemia include low levels of vitamin D, maternal hyperparathyroidism, hypomagnesemia, and primary hypoparathyroidism [8]. In our patient, the serum magnesium levels were in their normal ranges, and maternal hyperparathyroidism was ruled out as a likely cause of hypocalcemia. On further workup, the patient had very low levels of parathyroid hormone that caused low levels of serum calcium and high levels of serum phosphate that ultimately led to right-sided focal fits. The possibility of Di-George syndrome was ruled out as the patient had an intact parathyroid and thymus as visualized on the CT scan. To address the symptomatic hypocalcemia and increasing focal fits, treatment with alfacalcidol and calcium carbonate was initiated. Hussain S et al. also reported a similar case that presented with focal seizures and late-onset hypocalcemia due to hypoparathyroidism. Pertinently, their patient was treated with oral calcium supplements and cholecalciferol after which the seizures halted, and a six-month follow-up showed no complications [3]. Another study reported the successful treatment of a 28-day neonate by employing a similar therapeutic regimen [4]. The decision to initiate our treatment protocol was, therefore, based on evidence from these two cases [3-4]. In most instances, congenital hypoparathyroidism is linked with Di-George syndrome [9]. In our patient, however, Di-George syndrome was ruled out, as the CT showed a mediastinal window with an intact thymus and normal parathyroid glands in the neck.

Our case, therefore, highlights that prompt diagnosis of this idiopathic condition and appropriate management with calcium and vitamin D can help in preventing complications and mortality in neonates [3-4]. In order to better understand our case, we queried several databases. The results of our literature search are tabulated in Table 2.

**Table 2:** Previously documented cases of idiopathic primary hypoparathyroidism presenting as neonatal seizures

Case number	Case 1	Case 2
Authors	Hussain S et al.	LK P et al.
Age (days)	18 days	28 days
Sex	Male	Male
Calcium levels (mg/dL)	6.01 mg/dL	6.50 mg/dL
Parathyroid hormone levels	9.0 pg/ml	1.0 pg/ml
Birth history	Uneventful	Uneventful
Type of seizures	Focal	Generalized
Treatment given	Oral calcium supplement + Cholecalciferol	Calcium and Vitamin D3 supplements
Follow-up	Upon 6-month follow-up, the patient is seizure-free with no signs of nephrocalcinosis.	Upon 1-month follow up, the neonate is seizure-free.
Reference number	3	4

## Conclusions

Idiopathic primary hypoparathyroidism can rarely present as focal seizures in neonates that can be successfully treated with previously reported pharmaceutical regimens. These regimens typically consist of calcium and vitamin D3 supplements. However, the data pertaining to the utility and efficacy of such regimens remains strictly anecdotal. There is thus an unmet need for appropriate treatment guidelines to be curated for the management of similar cases in order to avoid complications and patient mortality.
